# Accurate identification of the six human *Plasmodium* spp. causing imported malaria, including *Plasmodium ovale wallikeri* and *Plasmodium knowlesi*

**DOI:** 10.1186/1475-2875-12-321

**Published:** 2013-09-13

**Authors:** Adriana Calderaro, Giovanna Piccolo, Chiara Gorrini, Sabina Rossi, Sara Montecchini, Maria Loretana Dell’Anna, Flora De Conto, Maria Cristina Medici, Carlo Chezzi, Maria Cristina Arcangeletti

**Affiliations:** 1 Unit of Microbiology and Virology, Department of Clinical and Experimental Medicine, Faculty of Medicine and Surgery, University of Parma, Viale A. Gramsci 14, Parma, Italy

**Keywords:** Imported malaria, Real-time PCR, Diagnosis

## Abstract

**Background:**

Accurate identification of *Plasmodium* infections in non-endemic countries is of critical importance with regard to the administration of a targeted therapy having a positive impact on patient health and management and allowing the prevention of the risk of re-introduction of endemic malaria in such countries. Malaria is no longer endemic in Italy where it is the most commonly imported disease, with one of the highest rates of imported malaria among European non-endemic countries including France, the UK and Germany, and with a prevalence of 24.3% at the University Hospital of Parma. Molecular methods showed high sensitivity and specificity and changed the epidemiology of imported malaria in several non-endemic countries, highlighted a higher prevalence of *Plasmodium ovale*, *Plasmodium vivax* and *Plasmodium malariae* underestimated by microscopy and, not least, brought to light both the existence of two species of *P. ovale* (*Plasmodium ovale curtisi* and *Plasmodium ovale wallikeri*) and the infection in humans by *Plasmodium knowlesi*, otherwise not detectable by microscopy.

**Methods:**

In this retrospective study an evaluation of two real-time PCR assays able to identify *P. ovale wallikeri*, distinguishing it from *P. ovale curtisi*, and to detect *P. knowlesi*, respectively, was performed applying them on a subset of 398 blood samples belonging to patients with the clinical suspicion of malaria.

**Results:**

These assays revealed an excellent analytical sensitivity and no cross-reactivity *versus* other *Plasmodium* spp. infecting humans, suggesting their usefulness for an accurate and complete diagnosis of imported malaria. Among the 128 patients with malaria, eight *P. ovale curtisi* and four *P. ovale wallikeri* infections were detected, while no cases of *P. knowlesi* infection were observed.

**Discussion and conclusions:**

Real-time PCR assays specific for *P. ovale wallikeri* and *P. knowlesi* were included in the panel currently used in the University Hospital of Parma for the diagnosis of imported malaria, accomplishing the goal of adhering to the recommendations of the World Health Organization to countries that are malaria-free to include the improvement of the early diagnosis of all cases of imported malaria.

## Background

Malaria, while the major cause of morbidity and mortality in adults and children worldwide [1] in endemic regions, is no longer endemic in Italy where it is the most commonly imported disease [2-4], with one of the highest rates of imported malaria among European non-endemic countries including France, the UK and Germany [5]. In the last decade, the routine use of molecular methods has transformed the epidemiology of malaria: in particular they enabled the identification of significantly more infections caused by *Plasmodium* species other than *Plasmodium falciparum* and of more mixed infections than detected by microscopy; molecular methods also highlighted both the existence of two distinct non-recombining species of *Plasmodium ovale* (classic type *Plasmodium ovale curtisi* and variant type *Plasmodium ovale wallikeri*) [6], and the infection in humans by *Plasmodium knowlesi*[7,8].

Different studies on *P. ovale* reported that *P. ovale wallikeri* is not confined to Southeast Asia but it circulates in African communities and that the two *P. ovale* species are generally sympatric in the countries where they occur [2,6].

On the other hand, *P. knowlesi*, that naturally occurs in macaques inhabiting forested areas of Southeast Asia [8], has developed the ability to naturally infect humans so that it is now recognized as the fifth species of *Plasmodium* causing malaria in humans [9,10] where it causes a spectrum of disease and where it can be fatal if not treated promptly [9,11,12]. *Plasmodium knowlesi* is widespread in humans in Malaysian Borneo [13-16] and the detection of *P. knowlesi* infections in travellers from Southeast Asia has been increasing [17] due to the availability of specific molecular assays able to reveal them. In particular, in Europe a few cases were described in Sweden [18], in The Netherlands [19], in Spain [20], and in Finland [21], but until now no data are available about imported *P. knowlesi* malaria cases in Italy. Therefore, *Plasmodium knowlesi* malaria should be considered in the differential diagnosis of any febrile traveller returning from forested areas of Southeast Asia [22]. The morphological resemblance of early trophozoites of *P. knowlesi* to *P. falciparum* and later erythrocytic stages of *Plasmodium malariae* makes it extremely difficult to identify *P. knowlesi* infections by microscopy only [15], emphasizing the need for the application of specific molecular assays [2,3,7].

In the light of these emerging epidemiological features, the molecular methods for the diagnosis of malaria caused by *P. falciparum*, *Plasmodium vivax*, *P. ovale*, and *P. malariae* in the laboratory located in the tertiary-care University Hospital of Parma [2,23,24] were recently implemented with real-time PCR assays able to identify *P. ovale wallikeri*, to distinguish it from *P. ovale curtisi*, and to detect *P. knowlesi*, respectively [2,7].

In this retrospective study an evaluation of these two latter molecular assays and the application of them on a subset of 398 blood samples were performed. Samples were chosen on the basis of broadly reflecting the epidemiological picture of imported malaria at the University Hospital of Parma (a higher prevalence of infections by *P. falciparum*, followed by *P. ovale*, *P. vivax* and *P. malariae*) and including all the samples available in the laboratory belonging to patients coming from Southeast Asia and that could therefore have an infection by *P. knowlesi*. This was done also in order to reveal the potential presence of mixed infections, including *P. ovale wallikeri* or *P. knowlesi*, otherwise undiagnosed, among the blood samples tested in the study and belonging to patients with clinical suspicion of malaria.

## Methods

### Samples

A subset of 398 blood samples collected between 2000 and 2012 at the University Hospital of Parma from 398 patients presenting on admission with signs and symptoms consistent with malaria was used. Countries of origin/visit of the 398 patients were: Africa (269 patients), Southeast Asia (25 patients), and South America (14 patients). For 81 patients the origin was unknown. The remaining nine patients referred no recent travel to endemic areas. The samples analyzed in this and previous studies had been obtained by the University Hospital of Parma for routine diagnosis purposes, as such no approval by the local review committee was required because of the laboratory diagnosis results had been reported in the medical records of the patients as a diagnostic answer to a clinical suspicion of malaria.

### Diagnostic methods

Microscopy was performed according to standard procedures as previously described [2,3,24]. Genomic DNA was purified as previously described [2] and subjected to routinely used molecular methods; an aliquot was stored at −20°C for further examination. According to the results of microscopic examination (negative or positive), the purified DNA were subjected to a genus-specific nested- or real-time PCR assay [25,26] or to species-specific nested- or real-time PCR assays for *P. falciparum*, *P. vivax*, *P. ovale curtisi*, and *P. malariae*, respectively, all targeting the 18S ribosomal RNA gene of *Plasmodium*[2,3,23].

The DNA purified from all 398 samples was subjected to real-time PCR assay for *P. knowlesi*[7] and that from 383 samples was subjected to real-time PCR assay for *P. ovale wallikeri* (for 15 among the 398 samples, the results of *P. ovale curtisi* and *P. ovale wallikeri* real-time PCR assays were previously reported [2]).

Each experiment included a negative control (a reaction mixture without DNA), and a positive control consisting of a synthetic *P. ovale wallikeri* target sequence. For specific real-time PCR, a synthetic DNA oligonucleotide containing a target sequence of *P. knowlesi* ssrRNA gene (synthesized by TIB Molbiol S.r.l., Genova, Italy) was used. Each blood sample negative for *Plasmodium* spp. was submitted to a Taq-Man based real-time PCR assay specific for the human β-actin gene as previously described [27], in order to assess both the success in DNA extraction and the absence of inhibitors of the DNA polymerase.

The analytical sensitivity and specificity of the real-time PCR assay specific for *P. ovale wallikeri* were previously assessed on a small number of samples [2]. In the present study, the specificity of this real-time PCR assay *versus* species of the genus *Plasmodium* other than *P. ovale wallikeri* was tested by analysing the samples positive for the other human plasmodial species included in the 398 samples assayed.

The detection limit of the *P. knowlesi* real-time PCR assay was determined by analysing in duplicate ten-fold dilutions in sterile double-distilled water of the synthetic *P. knowlesi* rDNA oligonucleotide, ranging from 50 × 10^12^ copies/μl to a theoretical value of 0.01 copies/μl. The analytical specificity of the new *P. knowlesi* real-time PCR was tested using genomic DNA samples from *in vitro* cultures of blood protozoa other than *Plasmodium* spp.*,* such as *Toxoplasma gondii* and *Leishmania infantum*. The specificity of the *P. knowlesi* real-time PCR assay *versus* species of the genus *Plasmodium*, other than *P. knowlesi,* was tested by analysing the samples positive for the other human plasmodial species included in the 398 tested in this study. Each real-time PCR run was performed testing all the samples, including controls, in duplicate.

## Results

Microscopy was positive for the presence of malaria parasites in 126 cases (31.7%) of the 398 samples analysed (Table 1): 101 *P. falciparum* (80.2%), nine *P. vivax* (7.1%), seven *P. ovale* (5.5%), one mixed infection *P. falciparum* + *P. ovale* (0.8%), and eight *Plasmodium* spp. (6.4%). The parasitaemia values ranged from <0.001 to 31.27% in the smears positive for *P. falciparum*, from 0.05 to 0.47% in the case of *P. vivax*, and from <0.01 to 1.18% in the case of *P. ovale* infections. In the *Plasmodium* spp. infections parasitaemia ranged from <0.001 to 0.6%. In the case of the mixed infection revealed by microscopy the parasitaemia was 4%. The highest parasitaemia value (31.27%) was observed in the blood smear belonging to a 43-year old African patient infected with *P. falciparum* presenting with fever, jaundice and thrombocytopaenia. The remaining 272 samples (68.3%) were negative by microscopy (Table 1).

**Table 1:** Different species of Plasmodia as detected by microscopy and by real-time PCR assays

**Microscopy**	**Real-time PCR assays**
	***Pf/Po/Poc/Pm/Pv/Pow/Pk***
101 *Pf*	99 *Pf*
	1 *Po*
	1 mixed *Pf* + *Poc + Pm*
9 *Pv*	7 *Pv*
	1 *Poc*
	1 *Pm*
7 *Po*	5 *Poc*
	1 *Pow*
	1 *Po*
1 mixed *Pf* + *Po*	1 *Pf*
8 *Plasmodium* spp.	2 *Pf*
	2 *Pow*
	1 *Poc*
	1 mixed *Pf* + *Pm*
	1 mixed *Pf* + *Pow*
	1 mixed *Pf* + *Po*
2 negative	1 *Pf*
	1 *Pm*
270 negative	270 negative

*PfP. falciparum*, *PoP. ovale* (in these cases the *P. ovale curtisi* and *P. ovale wallikeri* specific real-time PCR assays were not performed due to the unavailability of residual original sample), *PocP. ovale curtisi*, *PmP. malariae*, *PvP. vivax*, *PowP. ovale wallikeri*, *PkP. knowlesi*.

The real-time PCR assays revealed *Plasmodium*-specific DNA in 128 cases (32.1%) of the 398 samples analysed (Table 1): 103 *P. falciparum* (80.5%), seven *P. vivax* (5.5%), seven *P. ovale curtisi* (5.5%), three *P. ovale wallikeri* (2.4%), two *P. ovale* (1.6%), two *P. malariae* (1.6%), one mixed infection *P. falciparum* + *P. ovale wallikeri* (0.8%), one *P. falciparum* + *P. ovale curtisi* + *P. malariae* (0.8%), one *P. falciparum* + *P.ovale* (0.8%), and one *P. falciparum* + *P. malariae* (0.8%). In the cases reported as *P. ovale* (three single infections and one mixed), *P. ovale curtisi* and *P. ovale wallikeri* specific real-time PCRs assays were not performed due to the unavailability of residual original samples. The cycle threshold (Ct) values for the samples positive for *P. ovale wallikeri* ranged from 30.30 to 45.19 for the three single infections and 34.01 for the mixed infection. The remaining 270 samples (67.9%) were negative by real-time PCR assays (Table 1).

**Figure 1:**
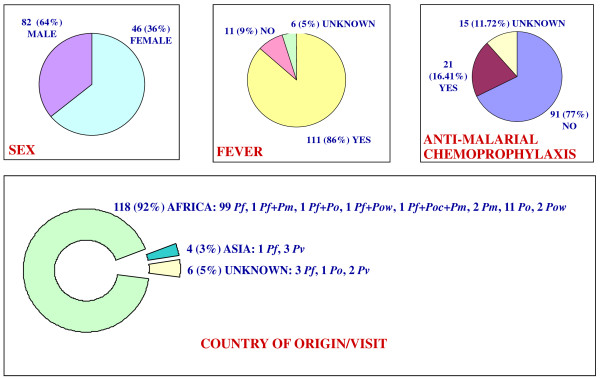
**Demographic data of the 128 patients with malaria diagnosed by PCR assays.***Pf* = *P. falciparum*; *Pv* = *P. vivax; Poc* = *P. ovale curtisi*; *Pow* = *P. ovale wallikeri*; *Pm* = *P. malariae*; *Pk = P. knowlesi*.

The *P. knowlesi*-specific real-time PCR assay gave a negative result for all 398 samples tested. The demographic data of all the 128 malaria cases regarding sex, fever, anti-malarial chemoprophylaxis, and country of origin/visit are shown in Figure 1. No cross-reactivity was detected with the *P. ovale wallikeri* real-time PCR assay when the 121 samples positive for different plasmodial species (*P. falciparum*, *P. malariae*, *P. ovale curtisi,* and *P. vivax*) were analysed, neither was a signal detected for any of the negative controls.

The detection limit of *P. knowlesi* real-time PCR assay was equal to a value of about ten copies/reaction. No cross-reactivity was detected with the *P. knowlesi* real-time PCR assay when the DNA purified from *T*. *gondii*, *L*. *infantum* and from the 128 samples positive for different plasmodial species (*P. falciparum*, *P. malariae*, *P. ovale curtisi*, *P*. *ovale wallikeri*, and *P. vivax*) were analysed, neither was a signal detected for any of the negative controls. All the negative samples for *Plasmodium* spp. were positive for the presence of the human β-actin gene.

## Discussion

Malaria is the most commonly imported disease in Italy as well as observed in Parma where its prevalence is about 24.3% (272 positive patients on a total of 1,117 patients presenting to University Hospital of Parma with the suspicion of malaria) [3]. The existence of two different species of *P. ovale* (*P. ovale curtisi* and *P. ovale wallikeri*) and the appearance in Europe of imported malaria cases by *P. knowlesi* make necessary the use of diagnostic assays able to detect them. Therefore, a panel of real-time PCR assays for the detection of *P. falciparum*, *P. vivax*, *P. ovale curtisi*, and *P. malariae* used at the University Hospital of Parma was implemented with a real-time PCR assay specific for *P. ovale wallikeri*, newly described by Calderaro et al. [2], and a real-time PCR assay for the detection of *P. knowlesi*, previously described in the literature [7]. The panel of real-time PCR assays currently used at the University Hospital of Parma for the diagnosis of malaria constitutes a robust system because it has been thoroughly validated in previous studies compared to reference molecular methods, including reference nested-PCR and genetic sequence analysis [23-25].

Regarding the *P. ovale wallikeri* real-time PCR assay, a brief evaluation including the detection limit and the analytical specificity previously made on a smaller number of samples was expanded by testing a greater amount of samples [2,5,8,23].

In this study, both the *P. ovale wallikeri* and *P. knowlesi* real-time PCR assays evaluated were proven to be very specific since no cross-reactivity was observed with the DNA obtained from the positive 128 samples containing other *Plasmodium* species, with the DNA obtained from blood protozoa other than *Plasmodium*, and with the DNA from the 270 negative samples for malaria (the veracity of which was guaranteed by the positivity of the β-actin internal control system and the negative results by the genus-specific PCR assays).

This finding is of particular importance regarding *P. knowlesi* given the inability of microscopy to diagnose infections by this species [15,17] which leads to the need for a thorough investigation to assess the correct prevalence of this parasite in the human population. On the contrary, some *P. knowlesi*-specific molecular methods previously described were reported to show cross-reactivity with other human *Plasmodium* spp. [28,29]. The 4/128 patients with malaria who came from Southeast Asia and the 6/128 of unknown origin, representing in this study the group of patients with the possibility of being infected with *P. knowlesi*, were all negative, highlighting in the population of patients tested the absence until now of cases of imported malaria by this species. The same result was obtained on 97 samples from patients without malaria coming from Southeast Asia or of unknown origin (that may, therefore, have come from Southeast Asia), already found to be negative by the genus-specific PCR assays. As expected, all the samples from African patients were negative for the presence of *P. knowlesi*. This assay showed in this study also an excellent analytical sensitivity, having a detection limit of about ten target copies/reaction, even if, while unfortunately, the clinical sensitivity of the assay could not be evaluated due to the lack of *P. knowlesi*-positive samples in the panel of samples analysed in the study.

This study, like many others already described in the literature [2,3,24,25,30], emphasizes once again the usefulness of molecular methods in the diagnosis of malaria as compared to the traditional microscopic investigation. The molecular assays revealed two additional single infections (one *P. falciparum* and one *P. malariae*) missed by microscopy, probably due to the very low presence of *Plasmodium* spp. [2,3,25]: the first case was a 26-year old Nigerian woman in Italy for one day with fever and abdominal pain for several days and the second was a 34-year old Ghanaian woman in Italy for five months with no clinical suspicion of malaria, but with fever after surgery for the removal of an abdominal and pelvic mass and subjected to a check for tropical diseases. Moreover, the molecular assays gave species identification in 6.3% (8/128) of the positive samples, revealing five single and three mixed infections in which plasmodial species had not been identified, limiting the results to genus identification (*Plasmodium* spp.). In such cases, the misdiagnosis by microscopy of the mixed infections can be due to the dominance of one parasite over the co-infecting one [3,31]. Microscopy did not reveal one mixed infection diagnosed by PCRs while one reported as a mixed infection by microscopy was not subsequently confirmed by molecular assays. Microscopy gave an incorrect diagnosis in 1.5% (2/128) of the positive cases, mistaking *P. vivax* with *P. ovale* and *P. malariae,* likely due to the fact that these infections do not reach as high parasite levels as those by *P. falciparum*.

**Figure 2:**
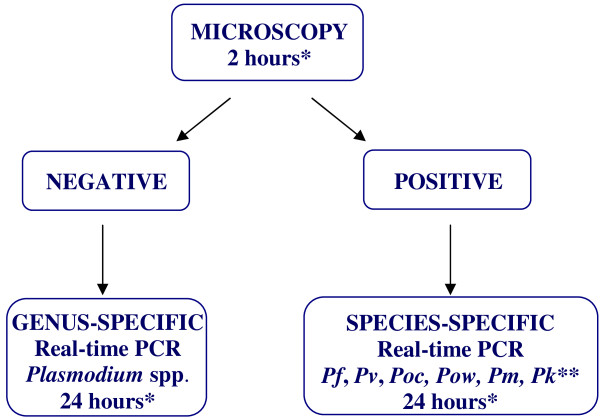
**Malaria diagnosis flow-chart.***Pf* = *P. falciparum*; *Pv* = *P. vivax; Poc* = *P. ovale curtisi*; *Pow* = *P. ovale wallikeri*; *Pm* = *P. malariae*; *Pk = P. knowlesi*. * time for producing the clinical report from the arrival of the sample; ** only in cases of patients from Southeast Asia.

Figure 2 shows the flow-chart currently adopted at the University Hospital of Parma for the diagnosis of imported malaria in the light of the results obtained in the present study.

## Conclusions

In this study the real-time PCR assays specific for *P. ovale wallikeri* and *P. knowlesi* were proven to be powerful diagnostic tools able to provide a novel insight into the epidemiology of malaria infections in a non-endemic area. Any meaningful investigations of the true epidemiology and biology of the two *P. ovale* variants, indistinguishable by microscopy, should include the application of sensitive and specific molecular methods of detection. Since the number of human infections by *P. knowlesi* is increasing, clinicians and laboratory personnel should be alert to this emerging species [32], and the potential cause of lethal malaria in humans, especially because it can be confused with the less dangerous species *P. malariae* by microscopic examination.

Real-time PCR assays specific for *P. ovale wallikeri* and *P. knowlesi* were included in the panel currently used in the University Hospital of Parma for the diagnosis of imported malaria, accomplishing the goal of adhering to the recommendations of the World Health Organization to countries that are malaria-free to include the improvement of the early diagnosis of all cases of imported malaria, among other specific programme objectives [33].

## Competing interests

The authors declare that there are no competing interests.

## Authors’ contributions

AC and GP conceived and designed the experiments. GP, CG, SM and SR performed the experiments. AC, GP, CG, SM, MLD, FDC, MCM, CC, and MCA analysed the data. AC contributed reagents/materials/analysis tools. AC, GP and CG wrote the paper. All authors read and approved the final manuscript.
